# A scoring system for predicting hepatocellular carcinoma risk in alcoholic cirrhosis

**DOI:** 10.1038/s41598-022-05196-w

**Published:** 2022-02-02

**Authors:** Kyunghan Lee, Gwang Hyeon Choi, Eun Sun Jang, Sook-Hyang Jeong, Jin-Wook Kim

**Affiliations:** 1 Department of Medicine, Seoul National University Bundang Hospital, 82, Gumi-ro 173 Beon-gil, Bundang-gu, Seongnam-si, Gyeonggi-do 13620 Republic of Korea; 2 Department of Internal Medicine, Seoul National University College of Medicine, Seoul, Republic of Korea

**Keywords:** Alcoholic liver disease, Hepatocellular carcinoma

## Abstract

The role of hepatocellular carcinoma (HCC) surveillance is being questioned in alcoholic cirrhosis because of the relative low HCC risk. This study aimed to assess the risk and predictors of HCC in Korean patients with alcoholic cirrhosis by using competing risk analysis. A total of 745 patients with alcoholic cirrhosis were recruited at a university-affiliated hospital in Korea and randomly assigned to either the derivation (n = 507) and validation (n = 238) cohort. Subdistribution hazards model of Fine and Gray was used with deaths and liver transplantation treated as competing risks. Death records were confirmed from Korean government databases. A nomogram was developed to calculate the Alcohol-associated Liver Cancer Estimation (ALICE) score. The cumulative incidence of HCC was 15.3 and 13.3% at 10 years for derivation and validation cohort, respectively. Age, alpha-fetoprotein level, and albumin level were identified as independent predictors of HCC and incorporated in the ALICE score, which discriminated low, intermediate, and high risk for HCC in alcoholic cirrhosis at the cut-off of 60 and 100. The risk of HCC can be stratified by using a combination of readily available clinical parameters (age, AFP level, and albumin level) in patients with alcoholic cirrhosis.

## Introduction

Alcohol-related liver disease (ALD) poses great global health burden. According to the Global Burden of Disease study 2017, 332,300 people died of ALD annually, which comprises approximately one fourth of mortalities associated with chronic liver disease^1^. Hepatocellular carcinoma (HCC), the most common form of primary liver cancer in ALD, is responsible for one-third of ALD-related mortality, and one-third of all HCC-related deaths are attributed to alcohol use globally^2^. Surveillance for HCC is recommended for high-risk groups in order to facilitate early detection and improve survival^3^. However, alcohol-related HCC is prone to insufficient surveillance and therefore delayed detection compared to viral hepatitis-associated HCC^4^. One of the reasons for under-surveillance may be related to relatively low incidence of HCC in ALD. For example, a recent Swedish cohort study (n = 3410) reported HCC incidence rate of 6.2 per 1000 person-years and the 10-year cumulative incidence of only 5.0% in alcoholic cirrhosis^5^, which was much lower than previously published (annual incidence of 2.6–2.9%)^6–9^. Another recent Danish study showed similar result (cumulative incidence of 6.0% after 10 years)^10^. These findings suggest that HCC screening for all alcoholic cirrhosis may not be cost-effective, and that further risk stratification is warranted to identify ideal candidates for surveillance in alcoholic cirrhosis.

In building a HCC prediction model, deaths and liver transplantations should be considered as competing events because many ALD patients experience hepatic decompensations and deaths before HCC is detected. Conventional Kaplan–Meier and Cox analysis may over-estimate the actual risk of HCC in the presence of competing risks^11^. For competing-risk survival analysis, cause-specific hazards or Fine-Gray model is recommended^12^. The aforementioned alcohol-related HCC prediction models, however, used conventional cox regression without competing risk analysis.

In this study, we sought to perform a competing-risk analysis for predicting the risk and predictors of HCC in alcoholic cirrhosis patients in Korea. For this aim, we linked the Korean national death registry data to hospital-based cohort data.

## Methods

### Study population and design

In this retrospective cohort study, an e-cohort was generated by using the clinical data warehouse of Seoul National University Bundang Hospital, a university-affiliated hospital in Korea^13–15^. The inclusion criteria were: 1) ALD based on ICD-10 code K70 AND presence of cirrhosis (see below), 2) > 20 years of age, 3) received baseline HCC screening by liver ultrasonography (US) with or without serum alpha-fetoprotein (AFP). The diagnosis of alcoholic cirrhosis was based on histology, endoscopic confirmation of varices or radiologic demonstration of cirrhosis. The exclusion criteria were 1) patients with short follow-up duration < 180 days, 2) patients with development of primary and secondary outcomes (see below) or other malignancies before or within 180 days from initial screening US, 3) serological positivity for hepatitis B or hepatitis C, 4) Child–Pugh class C patients at presentation. Child Pugh class C was excluded because HCC surveillance was generally not recommended unless they are on the transplant waiting list^3,16,17^.

The primary outcome was development of HCC. Secondary outcomes were liver transplantation and death which were assessed as competing risks. The death records were confirmed by using the Korean government database of vital statistics generated by Statistics Korea and Ministry of the Interior and Safety.

### HCC surveillance

All patients were advised to receive HCC surveillance which was comprised of liver US with or without serum AFP at 6–12 months of interval at the discretion of the attending hepatologists. Adherence to surveillance was operationally defined as at least yearly examination for liver ultrasound. Lack of adherence to surveillance included loss to follow-up. Multiphase CT or MRI were subsequently performed if liver US exam showed nodule(s) with a diameter ≥ 10 mm, or portal vein thrombosis, or increased AFP level. The diagnosis of hepatocellular carcinoma (HCC) was confirmed based on LiRAD 5 criteria^18^. Liver biopsy was performed to make a definitive diagnosis if imaging studies showed atypical findings^16^.

This study was approved by Seoul National University Bundang Hospital Institutional Review Board (IRB No: B-1907-553-105). All clinical investigations have been conducted according to the principles expressed in the Declaration of Helsinki. The requirement of informed consent was waived by Seoul National University Bundang Hospital Institutional Review Board due to the retrospective nature of this study and anonymous analysis of data.

### Statistical analysis

Enrolled patients were randomly assigned to one of two cohorts in a 2:1 ratio: the derivation and validation cohorts. Competing risk regression models were used with deaths and liver transplantations being treated as competing risks to assess the absolute risk of HCC and to identify the predictors of alcohol-related HCC from the derivation cohort. For competing risk analysis, the cause-specific cumulative incidences were plotted by non-parametric cumulative incidence function using STATA’s stcurve cif, and the subdistribution hazards model of Fine and Gray was built by using STATA’s stcrreg competing-risks regression^19,20^. Complete case analysis method was chosen for handling missing data. A nomogram was developed for calculating the HCC scoring system by using R rms package. The calibration of the scoring system was evaluated by using calibration curves (R riskRegression package). The predictive power and discriminative performance of the scoring system was compared with US-VA model^21^, an internally validated scoring system with age, sex, BMI, diabetes, platelet count, serum albumin, and serum AST/√ALT ratio as predictors, by using area under time-dependent ROC analysis with R timeROC package.

Continuous variables were expressed as their median values and interquartile range (IQR), and compared using Wilcoxon rank sum test. Categorical variables were expressed as percentages, and compared using chi-square test. All statistical analyses were performed using STATA for windows ver. 14 (STATA corp., Texas, USA) and R statistical package ver. 3.6.1 (The R Foundation for Statistical Computing, Vienna, Austria; http://R-project.org).

### Ethics approval

The IRB approved the study protocol (IRB No: B-1907-553-105).

### Consent to participate

Written consents were waived by the IRB due to the retrospective nature of study.

### Consent for publication

All authors agree to publication if the paper is accepted.

## Results

### Baseline characteristics of study cohorts

We identified 4980 patients with ALD who visited our institution and received screening US between April 1, 2004 and December 31, 2017. Among them, 745 patients with alcoholic cirrhosis were finally included in this study and randomly allocated to either derivation (n = 507) or validation cohort (n = 238). The baseline characteristics of the two cohorts were balanced without significant differences except for baseline AFP and GGT levels (Table 1). The adherence rate of HCC surveillance was 61.5%: 61.0% and 62.6% for derivation and validation cohort, respectively.

**Table 1:** Baseline characteristics of patients with alcoholic cirrhosis.

	Derivation cohort (n = 507)	Validation cohort (n = 238)	*P* value
Age (year)	56 (15)	55 (26)	0.444
Male sex (%)	79	76	0.288
Decompensated cirrhosis (%)	41	39	0.478
Diabetes (%)	33	32	0.826
Hypertension (%)	22	23	0.710
Dyslipidemia (%)	34	32	0.596
BMI (kg/m^2^)	25.2 (4.9)	25.0 (5.5)	0.856
Alcohol use (g/day)	54 (85)	54 (77)	0.894
Duration of alcohol use (y)	30 (20)	30 (20)	0.513
AFP (ng/mL)	3.8 (2.8)	4.1 (3.5)	0.015
AST (IU/L)	46 (51)	46 (52)	0. 276
ALT (IU/L)	36 (38)	33 (39)	0.830
Prothrombin time (INR)	1.08 (0.20)	1.09 (0.19)	0.549
Platelet count (× 10^3^/mm^3^)	175 (116)	170 (116)	0.563
Total bilirubin (mg/dL)	1.0 (0.9)	1.0 (0.9)	0.351
Albumin (mg/dL)	4.1 (0.8)	4.1 (0.8)	0.348
GGT (U/L)	111 (263)	165 (336)	0.047
ALP (U/L)	94 (61)	98 (65)	0.898
Child–Pugh class A/B (%)	66/34	66/34	0.850
FIB-4 index*	2.66 (3.68)	2.89 (3.60)	0.309
APRI score	0.71 (0.08)	0.78 (0.04)	0.317
Liver stiffness value, kPa	8.7 (11.3)	11.5 (9.2)	0.096

Continuous variables were expressed as their median values (interquartile range), and p-value was calculated using Wilcoxon rank-sum test. Categorical variables were expressed as absolute numbers (percentages), and p-value was calculated using chi-square test.

FIB-4 index^33^ = age (yr)xAST (U/L)/Platelet count (10^9^/L)x(ALT(U/L))^0.5^.

APRI score = AST(U/L)/platelet counts (10^9^/L)*0.4.

BMI, body mass index; HbsAg, hepatitis B surface antigen; Anti-HCV, antibody against hepatitis C virus; AFP, alpha-fetoprotein; AST, aspartate aminotransferase; ALT, alanine aminotransferase; INR, international normalized ratio; GGT, gamma-glutamyltransferase; ALP, alkaline phosphatase.

### Incidence of HCC in alcoholic cirrhosis

**Figure 1:**
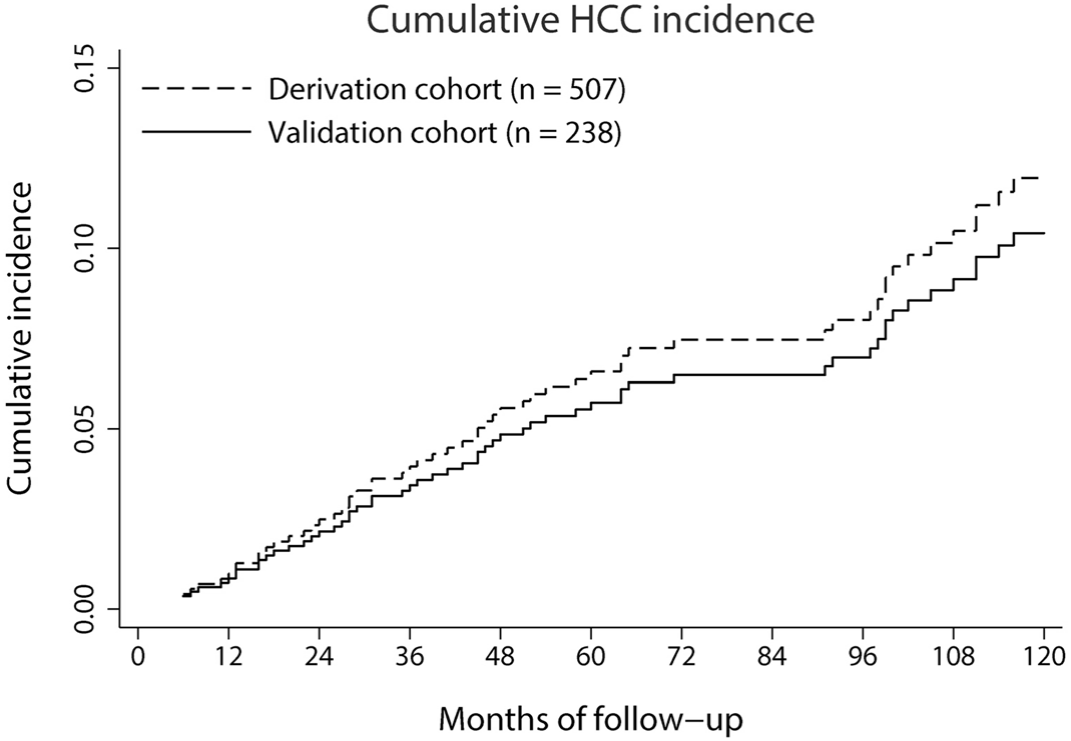
Cumulative incidence functions for HCC in the derivation and validation cohorts.

During the median follow-up period of 59 months (range 6–195), 62 patients developed HCC, 6 received liver transplantation, and 210 patients died without HCC. The cumulative HCC incidence was 7.0% and 6.1% at 5 years, and 15.3 and 13.3% at 10 years for derivation and validation cohort, respectively (Fig. 1).

### Predictors of HCC in alcoholic cirrhosis

Univariate subdistribution hazards model analysis of the derivation cohort demonstrated that older age, higher baseline AFP level, low serum albumin levels, and low platelet counts were significantly associated with increased risk of HCC. Among them, three predictors were independently identified through multivariate analysis: age, AFP level, and albumin level (Table 2). APRI and FIB-4 did not predict the HCC risks.

**Table 2:** Predictors for HCC development by Fine and Gray’s proportional subhazards model in derivation cohort (n = 507).

Variables	Univariate		Multivariate	
	Subhazard ratio (95% CI)	*P* value	Subhazard ratio (95% CI)	*P* value
Age (y)	1.04 (1.00–1.06)	0.012	1.03 (1.01–1.06)	0.033
Male sex	1.80 (0.71–4.58)	0.215		
Diabetes	1.11 (0.42–2.96)	0.834		
Hypertension	1.20 (0.64–2.26)	0.568		
Dyslipidemia	1.28 (0.71–2.34)	0.072		
BMI > 25 (kg/m^2^)	1.86 (0.95–3.64)	0.815		
Alcohol use (g/day)	1.00 (1.00–1.00)	0.978		
Duration of alcohol use (y)	1.01 (0.98–1.03)	0.61		
AFP (ng/mL, log10)	2.51 (1.18–5.32)	0.017	2.25 (1.07–4.74)	0.033
AST (IU/L)	1.00 (1.00–1.00)	0.220		
ALT (IU/L)	1.00 (1.00–1.00)	0.273		
Prothrombin time (INR)	1.46 (0.71–3.00)	0.307		
Platelet count (× 10^3^/mm^3^, log10)	0.19 (0.06–0.59)	0.004	0.36 (0.12–1.09)	0.070
Total bilirubin (mg/dL)	0.97 (0.83–1.14)	0.739		
Albumin (mg/dL, log10)	0.023 (0.005–0.099)	< 0.001	0.03 (0.01–0.69)	0.028
GGT (U/L)	1.00 (1.00–1.00)	0.268		
ALP (U/L)	1.00 (1.00–1.00)	0.637		
APRI score	0.98 (0.95–1.01)	0.229		
FIB-4	1.01 (0.99–1.03)	0.384		

^a^Patients with HbsAg or anti-HCV positivity.

### Development and validation of alcohol-associated liver cancer estimation (ALICE) scoring system

**Figure 2:**
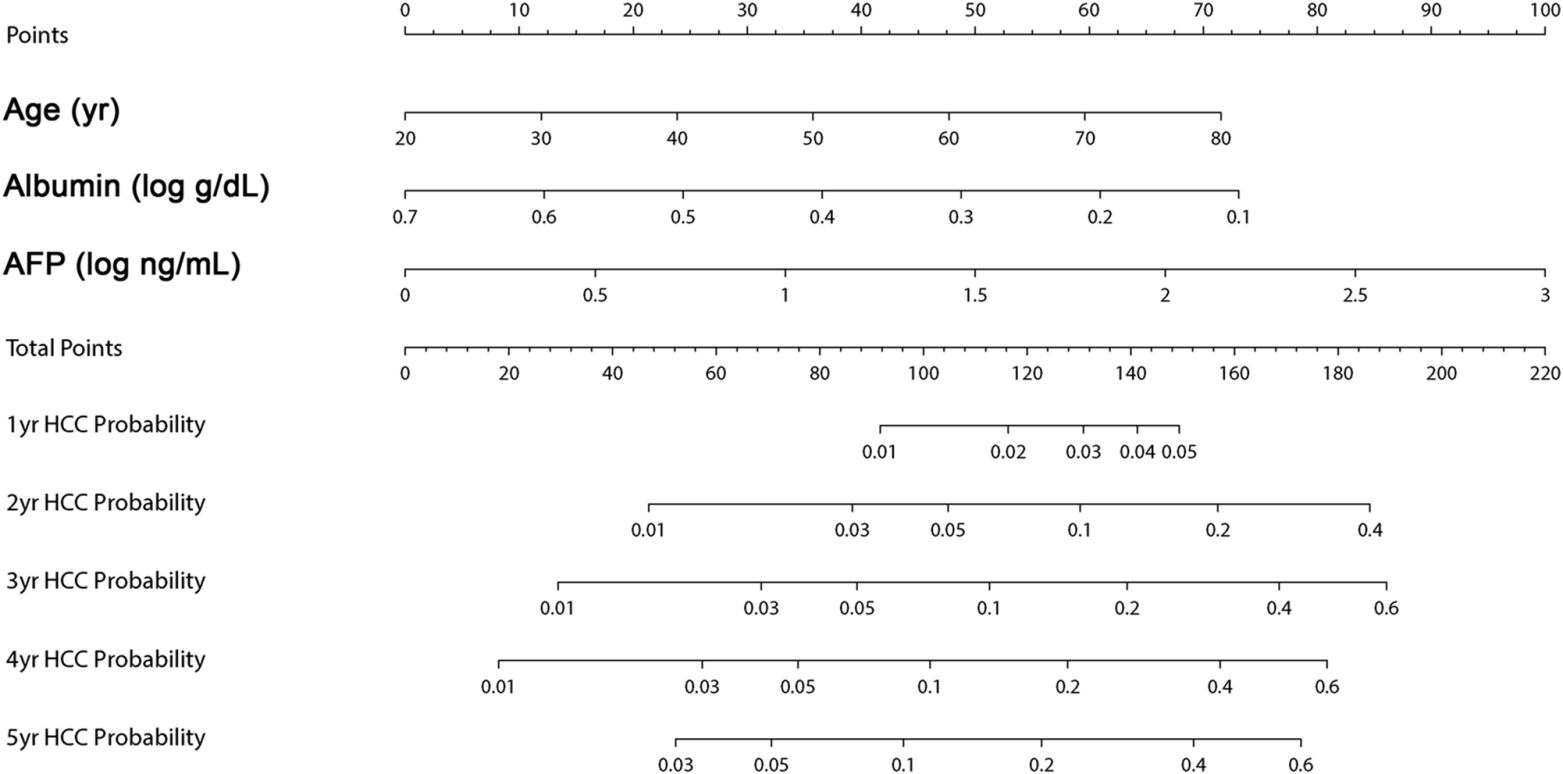
A nomogram for the alcohol-associated liver cancer estimation (ALICE) score.

**Figure 3:**
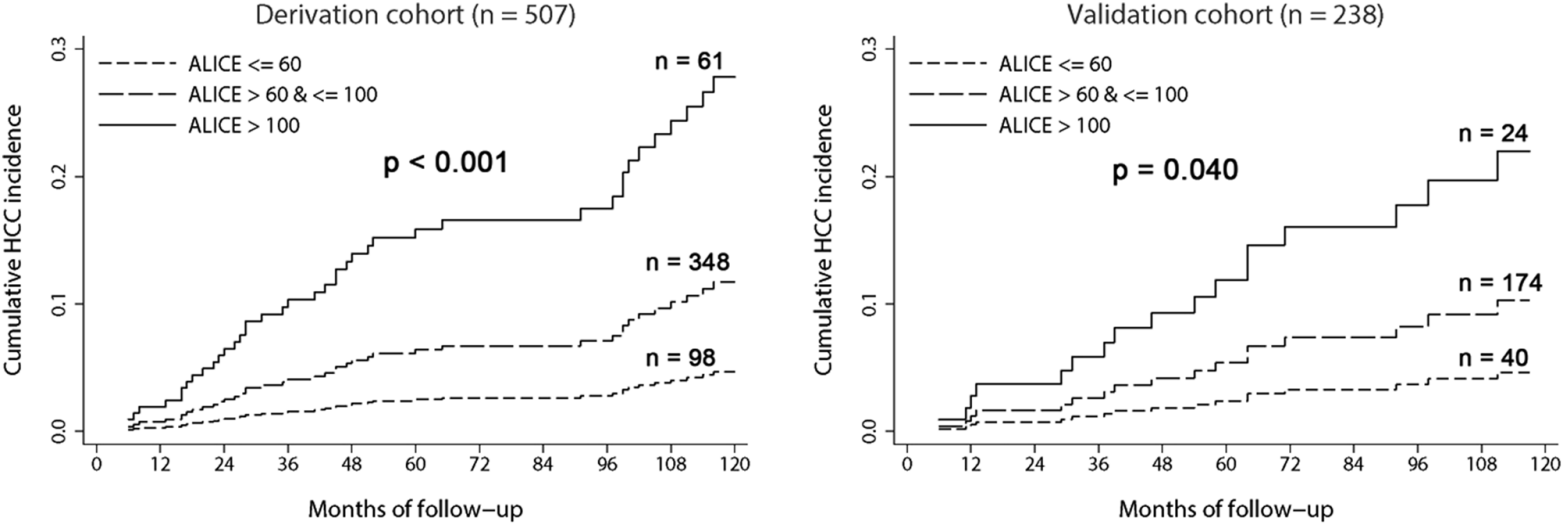
Cumulative incidence curves of HCC in the derivation and validation cohorts according to Alcohol-associated Liver Cancer Estimation (ALICE) score.

A parsimonious HCC prediction model, the alcohol-associated liver cancer estimation (ALICE) scoring system, was developed from the result of multivariate cumulative incidence function. Nomogram was constructed with three predictors to calculate the ALICE score (Fig. 2). The calibration plots of the nomogram showed good agreement between the observed and predicted HCC risks (Supplementary Fig. 1). When patients were stratified by ALICE score, HCC risk was minimal with a cut-off ≤ 60, whereas patients with a cut-off of > 60 and < 100 showed intermediate risk, and patients with ≥ 100 had highest risk for HCC (Fig. 3 and Table 3). The adherence rate of surveillance was higher in patients with high ALICE score: 52%, 62% and 74% for patients with ALICE score ≤ 60, > 60 and ≤ 100, and > 100, respectively in the overall patients (p = 0.004). When patients with early HCC development (within 1 year after enrollment), were excluded, the ALICE score was still able to stratify the risk of HCC (subhazard ratio = 2.56, 95% CI = 1.62–4.07; p < 0.001).

**Table 3:** Estimated cumulative incidence of HCC according to ALICE score.

ALICE score	Derivation cohort	Validation cohort
	**5-year HCC risk**	
≤ 60	2.4	2.4
> 60 and ≤ 100	6.4	5.4
> 100	15.9	11.9
	**10-year HCC risk**	
≤ 60	4.6	4.6
> 60 and ≤ 100	11.7	10.3
> 100	27.8	22.0

**Figure 4:**
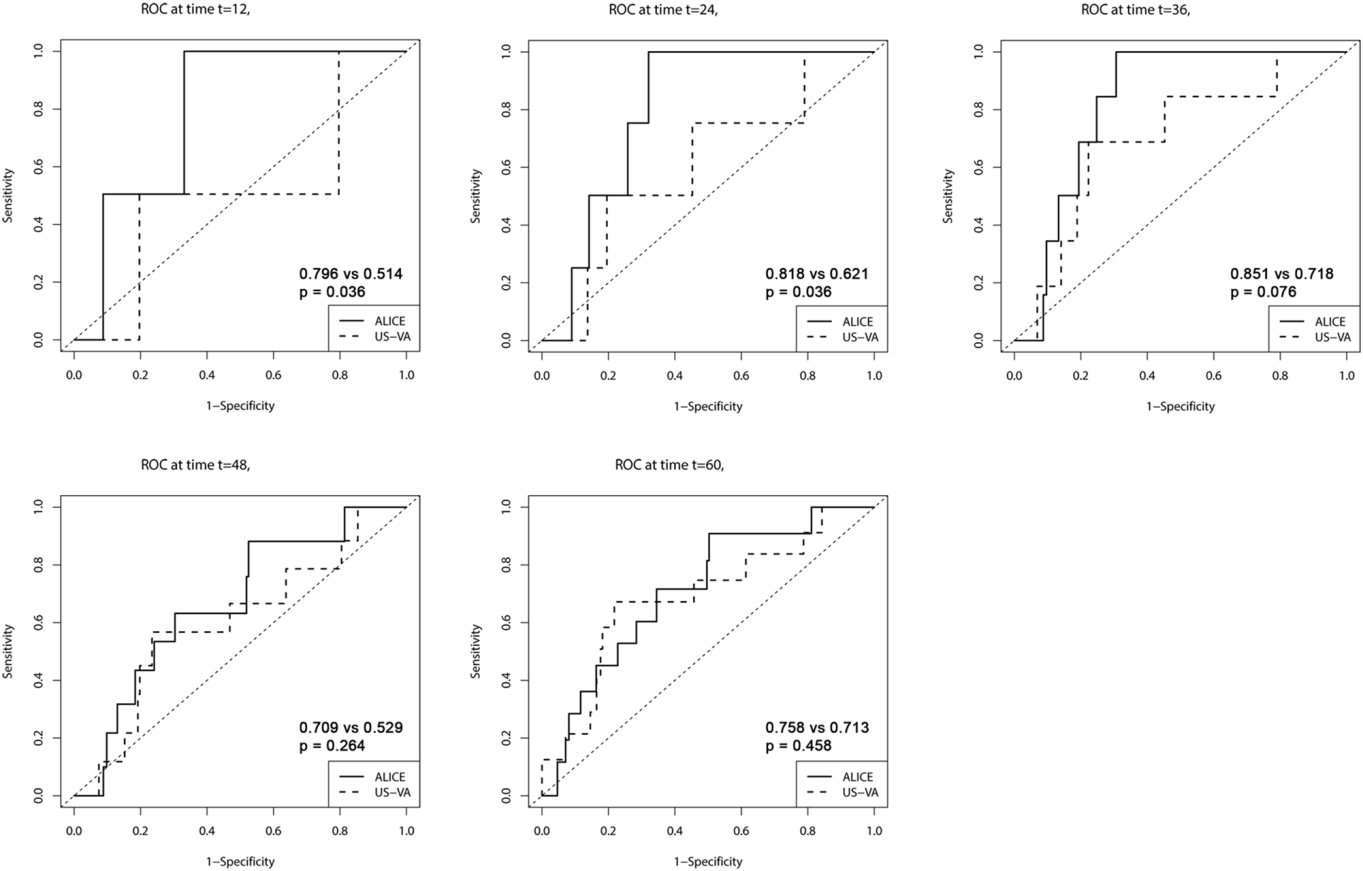
Comparison of time-dependent receiver operating characteristic curves between the ALICE score and US-VA score.

Finally, we compared the predictive performance of ALICE score with that of the US-VA model. Time-dependent ROC curve analysis revealed that the performance of ALICE score had comparable or higher AUC values than UA-VA score in the validation cohort (Fig. 4).

## Discussion

In this study, we assessed the HCC risk in Korean patients with alcoholic liver cirrhosis from a hospital-based cohort by using a competing-risk analysis with deaths and liver transplantations being treated as competing risks. The estimated cumulative HCC risk in our cohort was ~ 1.5% per year (Fig. 1). This incidence fell in the range between the two recent European studies (0.7% and 1.8%)^9,10^.

We also developed and internally validated a risk stratification model for HCC (*i.e.*, ALICE score). Compared to the recently developed prediction models^21,22^, we employed competing-risk analysis by incorporating mortality data from causes other than HCC. Liver cirrhosis is typically a multistate disease complicated by discrete outcomes^23^. If patients with competing outcomes such as non-HCC deaths are simply treated as right-censored cases, Kaplan Meier method may overestimate the real cumulative risks^23,24^. Moreover, the predicted risk of HCC does not necessarily correlate with the predicted rate by Cox model of HCC prediction^24^. Indeed, our cohort patients showed that censored cases due to non-HCC deaths were twice more than those censored due to HCC.

The role of HCC surveillance in alcoholic liver disease is still under debate. Practice guidelines recommend HCC surveillance in patients with cirrhosis due to alcohol and other etiologies on the ground that threshold HCC incidence of > 1.5%/year may justify cost-effectiveness of surveillance^3,16,25^. However, the “1.5%/year” cut-off itself has been doubted^26^. Since the risk of HCC in alcoholic cirrhosis may not be high enough to ensure cost-effectiveness^5,10^, risk stratification may be thus necessary to enhance the effectiveness of HCC surveillance in alcoholic cirrhosis.

We have built our risk stratification model based on three independent predictors of HCC risk: age, AFP level, and albumin level. AFP level was a significant predictor in addition to other well-established markers^21,22^, and this finding is in concordance with the French cohort study^9^. These three factors are readily available in routine practice, and nomogram-based ALICE score was able to discriminate the low, high, and super high-HCC risk groups in alcoholic cirrhosis. Patients with ALICE score ≤ 60 carries minimal risk for HCC and may not be indicated for routine HCC surveillance, whereas those with ≥ 100 show highest risk for HCC and regular surveillance may be justified. In other word, the ALICE score may serve dual purposes: (1) to exclude ALD patients with low risk from HCC surveillance, and (2) to identify patients with very high risk for HCC in need of enhanced surveillance. Further studies will be necessary to assess whether risk-based surveillance is cost-effective in alcoholic cirrhosis.

As mentioned earlier, competing risks were not considered in the US-VA model building. Time-dependent ROC analysis showed that the ALICE score had comparable or higher AUC values compared with the US-VA score (Fig. 4). Compared to the US-VA model, our score is more parsimonious with using only 3 readily available parameters. However, further validation would be warranted for the clinical utility of ALICE score by prospective studies.

It is of note that APRI and FIB-4 were not significant predictors of HCC in our data, because these non-invasive markers of hepatic fibrosis typically predict HCC risk in CHB^27^ and CHC^28^. This finding may be explained by the fact that the risk of HCC may be less dependent on the transaminase levels in alcoholic cirrhosis compared to viral hepatitis (Table 2). The pathogenetic mechanisms responsible for this observation needs to be further investigated in future studies.

The overall adherence rate to surveillance was 61.5%, which was slightly higher than the adherence rate of cirrhotic patients from a recent meta-analysis (52%)^29^. Of, the rate was lower (52%) in patients with low ALICE score ≤ 60. It can be speculated that attending physicians might have put less stress on the importance of surveillance in these seemingly low-risk patients. However, it cannot be ruled out that suboptimal surveillance may have underestimated the HCC incidence and further validation is needed.

There are potential limitations in our study. First, the study population is confined to Koreans. The performance of our model may need to be confirmed in other ethnic groups. Second, although we tried to minimize selection bias by using our pre-defined EMR query templates^13,30^, the nature of retrospective design suffers potential liability for bias. Third, our model has been validated only in an internal validation cohort, which is very similar to the derivation cohort. Further external validation is needed by prospective studies. Further cost-effectiveness analysis may also be needed for the clinical utility of ALICE score-guided surveillance strategy. Fourth, the diagnosis of cirrhosis was mostly made clinically, and there was a possibility that a portion of liver cirrhosis might have been excluded from our cohort^31,32^. Conversely, it might also be possible that some non-cirrhotic patients with acute exacerbation of portal hypertension had been selected in our study. Since liver biopsy is not generally required for the management of compensated alcoholic liver disease, however, we believe that our model can be applicable to real-world practice of clinically diagnosed alcoholic liver cirrhosis. Finally, we were not able to collect longitudinal drinking amount and its effect on portal hypertension or incidence of HCC. The revised version of our model may need to incorporate the current drinking vs. abstinence factor.

In conclusion, the risk of HCC can be stratified by using a combination of readily available clinical parameters (age, AFP level, and albumin level) in patients with alcoholic cirrhosis.

## Supplementary Information

Supplementary Information 1.

Supplementary Information 2.

## Data Availability

Data will be shared on request to the corresponding author with permission of our IRB.

## Abbreviations

AFP: Alpha-fetoprotein
ALICE: Alcohol-associated liver cancer estimation
ALD: Alcohol-related liver disease
ALP: Alkaline phosphatase
ALT: Alanine aminotransferase
AST: Aspartate aminotransferase
AUC: Area under the curve
BCLC: Barcelona Clinic Liver Cancer
GGT: Gamma-glutamyl transferase
HBV: Hepatitis B virus
BMI: Body mass index
HCC: Hepatocellular carcinoma
HCV: Hepatitis C virus
ICD: International Classification of Disease
IQR: Interquartile range
ROC: Receiver operating characteristic
INR: International normalized ratio
NAFLD: Non-alcoholic fatty liver disease
US: Ultrasonography
