# Supplementary material for: A scoring system for predicting hepatocellular carcinoma risk in alcoholic cirrhosis
Source: Sci Rep. 2022 Feb 2;12:1717. doi: 10.1038/s41598-022-05196-w (PMC8810867; doi:10.1038/s41598-022-05196-w)
Supplement: Supplementary file 1 — Supplementary Information 1. [file 41598_2022_5196_MOESM1_ESM.docx]

**A scoring system for predicting hepatocellular carcinoma risk in alcoholic cirrhosis**

**Supplementary Table 1.** Baseline characteristics of patients with alcoholic cirrhosis including patients with comorbid chronic viral hepatitis.

|  | Derivation cohort (n=643) | Validation cohort (n=322) | P value |
| --- | --- | --- | --- |
| Age (year) | 55 (15) | 55 (12) | 0.364 |
| Male sex (%) | 80 | 77 | 0.266 |
| Decompensated cirrhosis (%) | 37 | 33 | 0.137 |
| Diabetes (%) | 31 | 30 | 0.832 |
| Hypertension (%) | 21 | 23 | 0.589 |
| Dyslipidemia (%) | 33 | 32 | 0.796 |
| BMI (kg/m^2^) | 25.3 (4.8) | 25.2 (5.9) | 0.745 |
| Alcohol use (g/day) | 54 (89) | 54 (85) | 0.972 |
| Duration of alcohol use (y) | 30 (15) | 30 (20) | 0.598 |
| HBsAg positivity (%) | 19 | 21 | 0.464 |
| Anti-HCV positivity (%) | 3.4 | 5.0 | 0.242 |
| AFP (ng/mL) | 3.7 (3.2) | 4.1 (3.6) | 0.015 |
| AST (IU/L) | 45 (51) | 46 (48) | 0. 671 |
| ALT (IU/L) | 38 (42) | 36 (40) | 0.703 |
| Prothrombin time (INR) | 1.08 (0.17) | 1.08 (0.18) | 0.955 |
| Platelet count (x10^3^/mm^3^) | 173 (107) | 173 (107) | 0.822 |
| Total bilirubin (mg/dL) | 1.0 (0.7) | 1.0 (0.8) | 0.154 |
| Albumin (mg/dL) | 4.2 (0.7) | 4.1 (0.7) | 0.369 |
| GGT (U/L) | 97 (117) | 101 (280) | 0.176 |
| ALP (U/L) | 90 (57) | 91 (57) | 0.801 |
| Child-Pugh class A / B (%) | 70/30 | 72/30 | 0.439 |
| FIB-4 index* | 2.56 (3.40) | 2.44 (4.63) | 0.872 |
| APRI score |  |  |  |
| Liver stiffness value, kPa | 7.9 (8.7) | 8.7 (8.4) | 0.732 |

Continuous variables were expressed as their median values (interquartile range), and p-value was calculated using Wilcoxon rank-sum test. Categorical variables were expressed as absolute numbers (percentages), and p-value was calculated using chi-square test.

FIB-4 index^1^ = age (yr)ｘAST (U/L)/Platelet count (10^9^/L)ｘ(ALT(U/L))^0.5^

APRI score = AST(U/L)/platelet counts (10^9^/L)*0.4

BMI, body mass index; HbsAg, hepatitis B surface antigen; Anti-HCV, antibody against hepatitis C virus; AFP, alpha-fetoprotein; AST, aspartate aminotransferase; ALT, alanine aminotransferase; INR, international normalized ratio; GGT, gamma-glutamyltransferase; ALP, alkaline phosphatase

**Supplementary Table 2.** Predictors for HCC development by Fine and Gray’s proportional subhazards model in derivation cohort including patients with comorbid chronic viral hepatitis (n = 643)

| **Variables** | | **Univariate** | | **Multivariate** | |
| --- | --- | --- | --- | --- | --- |
|  | Subhazard ratio **(95% CI)** | | ***P value*** | Subhazard ratio **(95% CI)** | ***P value*** |
| Age (y) | 1.02 (1.00-1.04) | | **0.03** | 1.03 (1.01-1.05) | **< 0.01** |
| Male sex | 2.58 (1.03-6.44) | | **0.04** | 2.48 (0.99-6.22) | 0.05 |
| Diabetes | 0.83 (0.40-1.72) | | 0.62 |  |  |
| Hypertension | 1.35 (0.81-2.26) | | 0.25 |  |  |
| Dyslipidemia | 1.20 (0.73-1.97) | | 0.47 |  |  |
| BMI (kg/m^2^) | 1.03 (0.98-1.09) | | 0.22 |  |  |
| Alcohol use (g/day) | 1.00 (1.00-1.00) | | 0.86 |  |  |
| Duration of alcohol use (y) | 1.01 (0.98-1.03) | | 0.61 |  |  |
| HBV/HCV positive^a^ | 1.71 (1.02-2.85) | | **0.04** | 1.80 (1.03-3.12) | **0.04** |
| AFP (ng/mL, log10) | 2.30 (1.48-3.57) | | **< 0.01** | 1.76 (1.10-2.82) | **0.02** |
| AST (IU/L) | 1.00 (1.00-1.00) | | 0.19 |  |  |
| ALT (IU/L) | 1.00 (1.00-1.00) | | 0.53 |  |  |
| Prothrombin time (INR) | 1.70 (0.93-3.13) | | 0.08 |  |  |
| Platelet count (x10^3^/mm^3^) | 0.99 (0.99-1.00) | | **< 0.01** | 0.99 (0.99-1.00) | **0.02** |
| Total bilirubin (mg/dL) | 0.99 (0.90-1.10) | | 0.90 |  |  |
| Albumin (mg/dL) | 0.66 (0.44-1.00) | | 0.05 |  |  |
| GGT (U/L) | 1.00 (1.00-1.00) | | 0.14 |  |  |
| ALP (U/L) | 1.00 (1.00-1.00) | | 0.99 |  |  |
| APRI score | 0.99 (0.96-1.02) | | 0.36 |  |  |
| FIB-4 | 1.01 (0.99-1.03) | | 0.29 |  |  |

^a^Patients with HbsAg or anti-HCV positivity.

**Supplementary Table 3.** Estimated cumulative incidence of HCC according to ALICE score in alcoholic cirrhosis patients including comorbid chronic viral hepatitis

| ALICE score | Derivation cohort | Validation cohort |
| --- | --- | --- |
|  | 5-year HCC risk | |
| <= 120 | 0.9 | 1.8 |
| >120 and <=180 | 7.2 | 7.0 |
| >180 | 31.5 | 33.8 |
|  | 10-year HCC risk | |
| <= 120 | 1.3 | 2.8 |
| >120 and <=180 | 17.0 | 16.6 |
| >180 | 37.2 | 39.9 |

**Supplementary Figure Legend**

**Supplementary Fig. 1.** Calibration plot of ALICE score nomogram for 5-year risk of HCC in derivation and validation cohort. X-axis denotes the nomogram-predicted risk and y-axis denotes actual risk.

Uncategorized References

1 Sterling, R. K., Lissen, E. *et al.* Development of a simple noninvasive index to predict significant fibrosis in patients with HIV/HCV coinfection. *Hepatology* **43**, 1317-1325, doi:10.1002/hep.21178 (2006).
