# Supplementary figures and images for: A scoring system for predicting hepatocellular carcinoma risk in alcoholic cirrhosis
Source: Sci Rep. 2022 Feb 2;12:1717. doi: 10.1038/s41598-022-05196-w (PMC8810867; doi:10.1038/s41598-022-05196-w)

**A**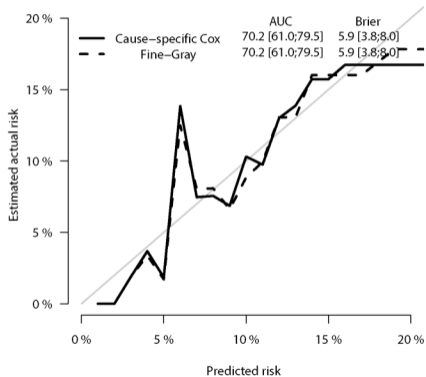**B**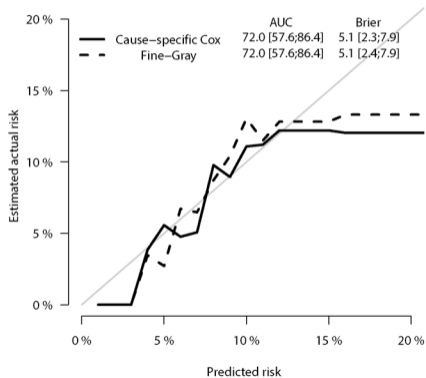

Supplement: Supplementary file 2 — Supplementary Information 2. [file 41598_2022_5196_MOESM2_ESM.pdf]
